# Synthesis, crystal structure and Hirshfeld surface analysis of a propyl 4-{[1-(2-methyl-4-nitro­phen­yl)-1*H*-1,2,3-triazol-4-yl]meth­oxy}benzoate copper(II) chloride complex

**DOI:** 10.1107/S2056989025001732

**Published:** 2025-03-04

**Authors:** Muminjon Hakimov, Ilkhomjon Ortikov, Tulkinjon Sattarov, Bakhodir Tashkhodjaev, Akmaljon Tojiboev

**Affiliations:** a Namangan State University, Boburshoh str. 161, Namangan, 160107, Uzbekistan; b Alfraganus University, Yukari Karakamysh str. 2A 100190, Tashkent, Uzbekistan; c Institute of the Chemistry of Plant Substances, Uzbekistan Academy of Sciences, Mirzo Ulugbek Str. 77, Tashkent 100170, Uzbekistan; d University of Geological Sciences, Olimlar Str. 64, Tashkent 100170, Uzbekistan; Illinois State University, USA

**Keywords:** crystal structure, copper (II) complex, 1,2,3-triazole, hydrogen bonding, Hirshfeld surface analysis

## Abstract

The core of the title complex, di­chlorido­bis­(propyl 4-{[1-(2-methyl-4-nitro­phen­yl)-1*H*-1,2,3-triazol-4-yl]meth­oxy}benzoate)copper(II), [CuCl_2_(C_20_H_20_N_4_O_5_)_2_], which belongs to the copper(II) complex family, consists of two C_20_H_20_N_4_O_5_ ligands and two chloride ligands arranged around the metal, forming a *trans*-di­chlorido square-planar complex. In the crystal, the mol­ecules are linked by C—H⋯Cl and C—H⋯O hydrogen bonds as well as by aromatic π–π stacking inter­actions into a three-dimensional network. To further analyse the inter­molecular inter­actions, a Hirshfeld surface analysis was performed.

## Abstract 2

The title coordination compound was synthesized upon complexation of propyl 4-{[1-(2-methyl-4-nitro­phen­yl)-1*H*-1,2,3-triazol-4-yl]meth­oxy}benzoate and copper(II) chloride at 323 K. It crystallizes as a centrosymmetric dimer, with one copper atom, two chlorine atoms and two propyl 4-{[1-(2-methyl-4-nitro­phen­yl)-1*H*-1,2,3-triazol-4-yl]meth­oxy}benzoate ligands in the asymmetric unit.

## 1. Chemical context

Transition-metal halides may be reacted with functionalized organic mol­ecules (for example carb­oxy­lic acids, amides or amines) to produce neutral or ionic coordination compounds that combine and leverage the properties of both components (Constable *et al.*, 2021 ▸). 1,2,3-Triazoles comprise an inter­esting class of heterocyclic compounds (Bozorov *et al.*, 2019 ▸), and the synthesis of ligand-based 3*d* metal complexes from these compounds is of even greater inter­est (Dheer *et al.*, 2017 ▸). The discovery by Sharpless and coworkers in 2001 (Kolb *et al.*, 2001 ▸) of click chemistry, especially the copper-catalysed alkyne-azide cyclo­addition (CuAAC) methodology for the preparation of triazole derivatives, has accelerated important advances in many scientific areas. This copper-catalysed process constituted a substantial development on the classical Huisgen-type thermal 1,3-dipolar cyclo­addition as it permitted the regioselective preparation of 1,4- and 1,5-disubstituted 1,2,3-triazoles (Huisgen, 1963 ▸; Ling *et al.*, 1996 ▸; Hein & Fokin, 2010 ▸; Liang & Astruc, 2011 ▸). Daniel Mendoza and co-worker reported the new copper(II) complexes supported by 2-mercapto and 4-mercapto­pyridine-derived 1,2,3-triazole ligands. Their new complexes were tested in the CuAAC process under a variety of reaction conditions. The overall catalytic data demonstrated these complexes displayed the best CuAAC performance in alcoholic solvents without the need for an external reducing agent (Gonzalez-Silva *et al.*, 2019 ▸). Herein, we report the synthesis of the coordination compound, **1**, formed from propyl 4-{[1-(2-methyl-4-nitro­phen­yl)-1*H*-1,2,3-triazol-4-yl]meth­oxy}benzoate and copper(II) chloride and examined it using single-crystal X-ray diffraction and Hirshfeld surface studies as a part of our ongoing inter­est in 1,2,3-triazole derivatives, a continuation of our recently published work on the synthesis of triazole derivatives (Hakimov *et al.*, 2024 ▸).
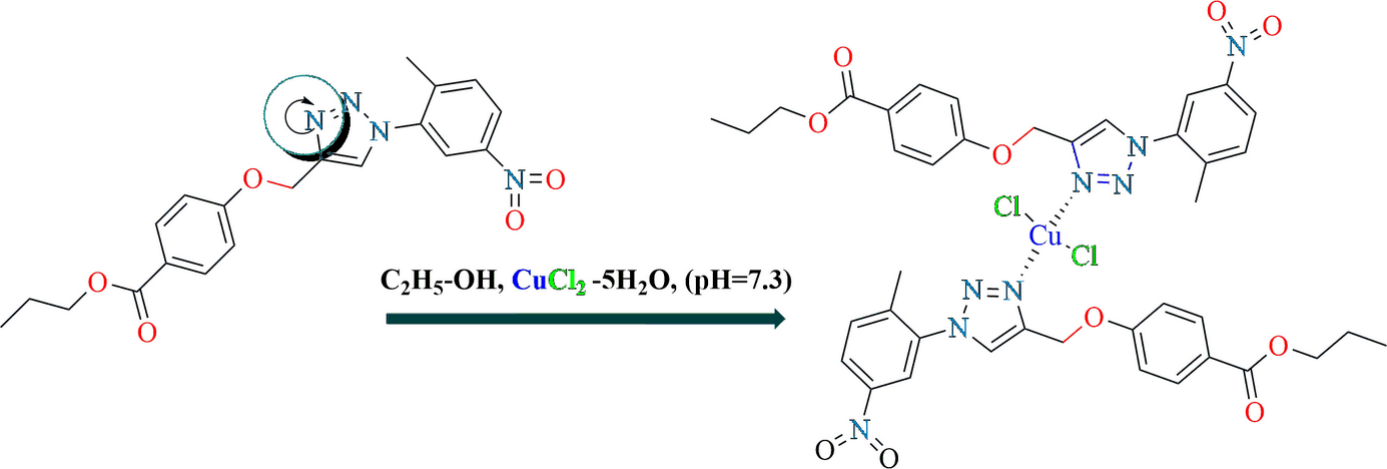


## 2. Structural commentary

**Figure 1:**
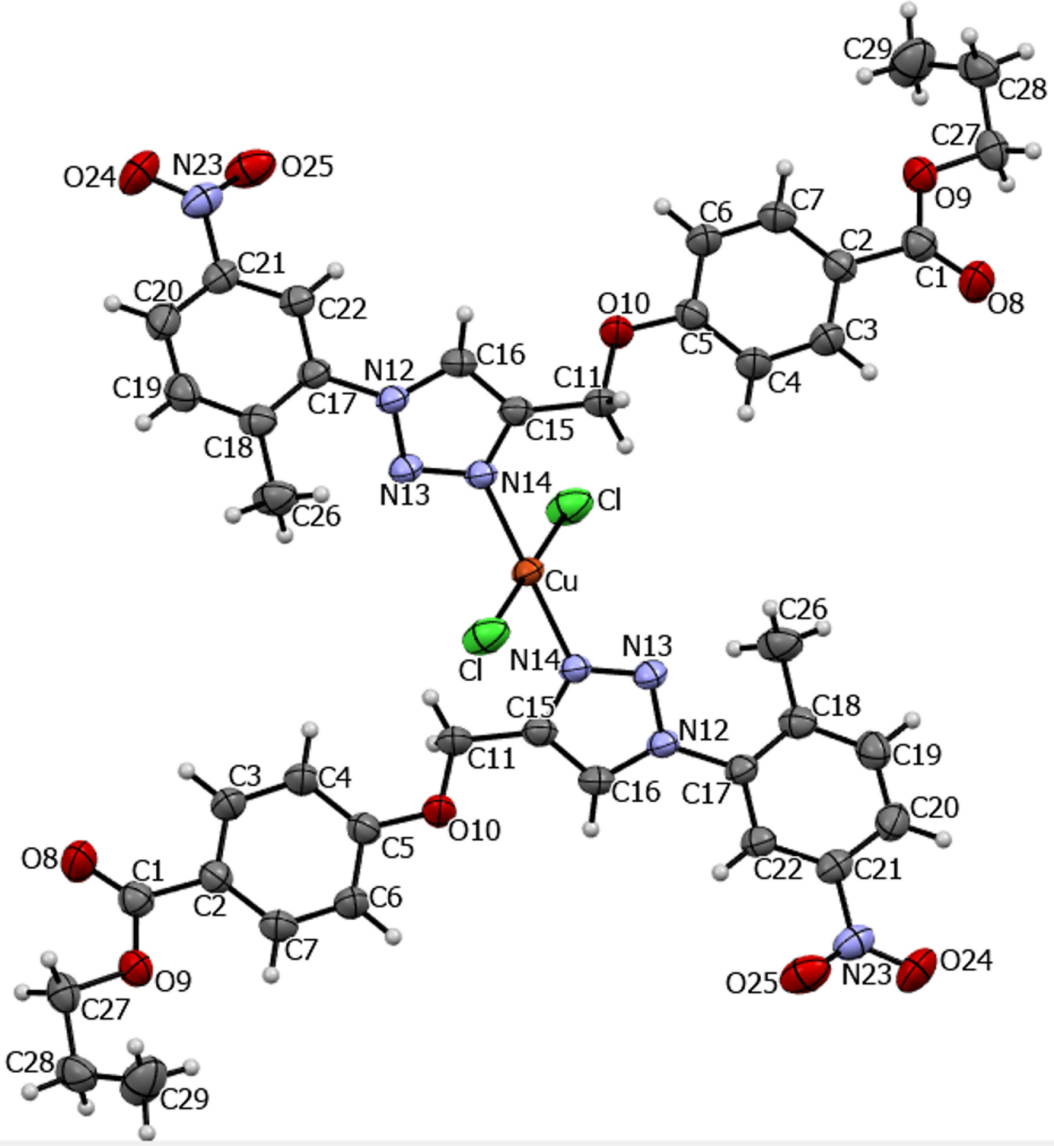
Ellipsoid plot of the title compound with displacement ellipsoids drawn at the 50% probability level.

Compound **1** crystallizes in the monoclinic space group *P*2_1_/*c*. Fig. 1 ▸ depicts a perspective view of the mononuclear centrosymmetric complex, [(Cu)(*L*)_2_(Cl)_2_], where *L* = propyl 4-{[1-(2-methyl-4-nitro­phen­yl)-1*H*-1,2,3-triazol-4-yl]meth­oxy}benzoate, with the atom-labeling scheme. The asymmetric unit contains half of the mol­ecule, with the copper atom coincident with an inversion center, which renders the two C_20_H_20_N_4_O_5_ ligands crystallographically equivalent. Likewise, the *trans*-chloride ligands are crystallographically equivalent. The copper(II) center is coordinated by a single nitro­gen of each of the two 1*H*-1,2,3-triazole ligands with an N14—Cu bond length of 2.009 (2) Å and to two chlorine atoms with a Cu—Cl distances of 2.2460 (9) Å. Inter­estingly, the O10 atoms are located far away from the Cu center [4.451 (2) Å], ruling out a possible bidentate coordination of each 1,2,3-triazole ligand for the title compound. The coordination of the Cu metal center adopts a square-planar geometry, with τ_4_ = 0 (Yang *et al.*, 2007 ▸). According to the structural data for the title compound, the torsion angles O10—C11—C15—C16 and C16—N12—C17—C22 of the triazole ring with neighboring atoms are 53.6 (5) and −47.3 (5)°, respectively.

## 3. Supra­molecular features

**Figure 2:**
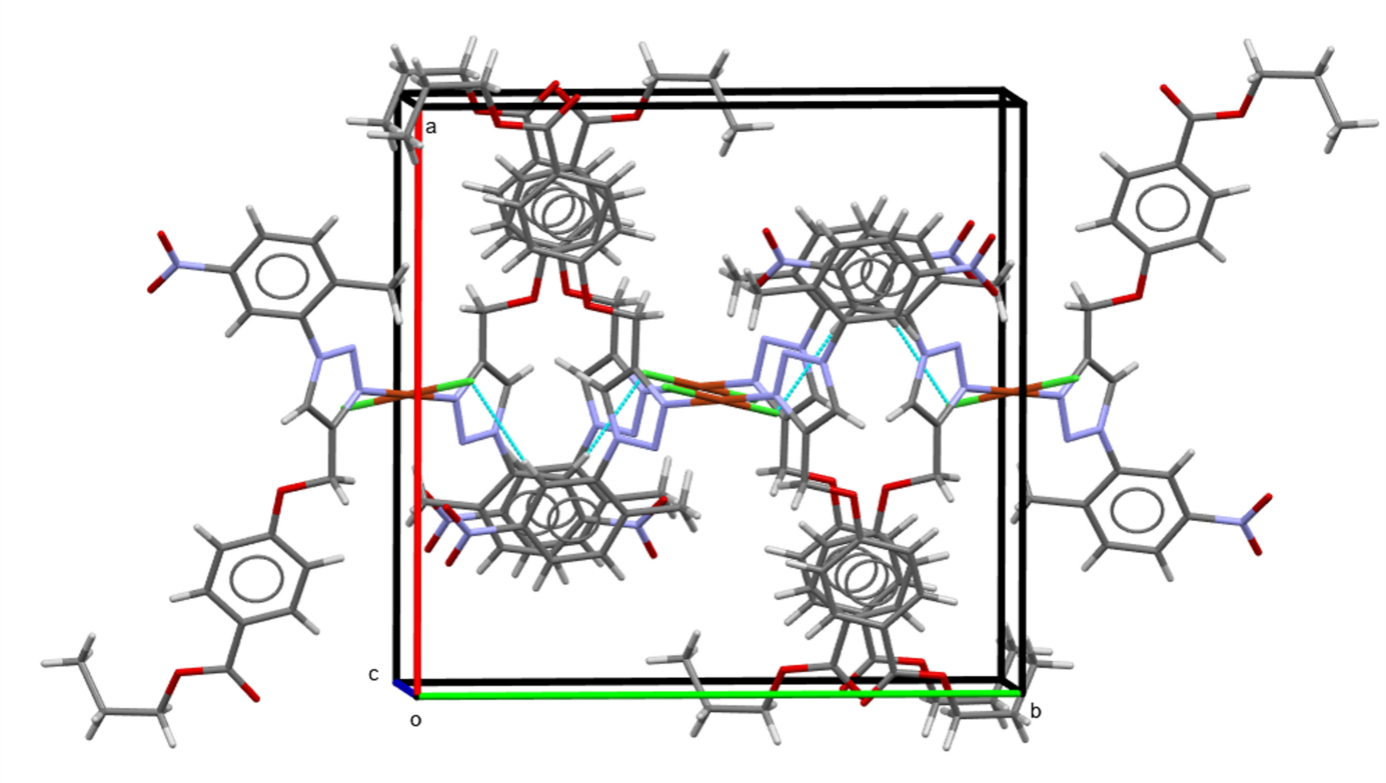
Crystal packing of the title compound. Hydrogen bonds are shown as blue dashed lines.

In the crystal structure of the title compound, no classical strong hydrogen bonds are observed. Some inter­mol­ecular C—H⋯O and C—H⋯Cl contacts (Table 1 ▸) can be identified as hydrogen bonds by Hirshfeld surface analysis (*vide infra*). For the complexes, a chain along the *c-*axis direction is observed due to stacking effects between the benzene rings (Fig. 2 ▸). These contacts link the mol­ecules into a three-dimensional network, complemented by short ring-inter­actions with stacking between the triazole (centroid *Cg*1), propyl benzoate (centroid *Cg2*) rings and 1-methyl-5-nitro­benzene (centroid *Cg3*) rings [*Cg*1⋯*Cg1′* = 5.5492 (18) Å, *Cg2*⋯*Cg2′* = 4.009 (2) Å and *Cg3*⋯*Cg3′* = 4.094 (2) Å, with slippages of 0.46, 1.799 and 2.091 Å, respectively].

## 4. Hirshfeld surface analysis

**Figure 3:**
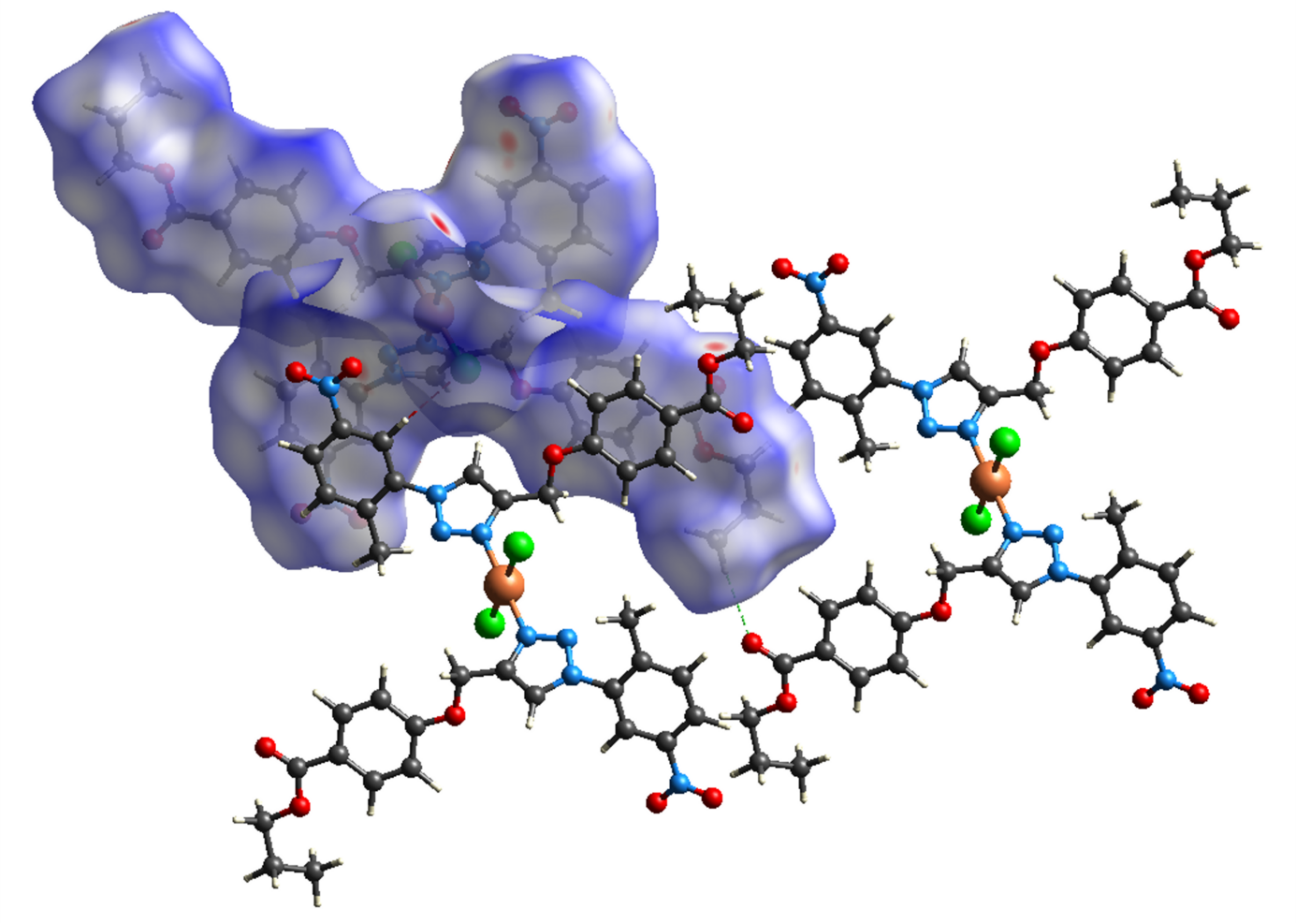
Hirshfeld surface of **1** mapped over *d*_norm_ and close inter­mol­ecular contacts.

**Figure 4:**
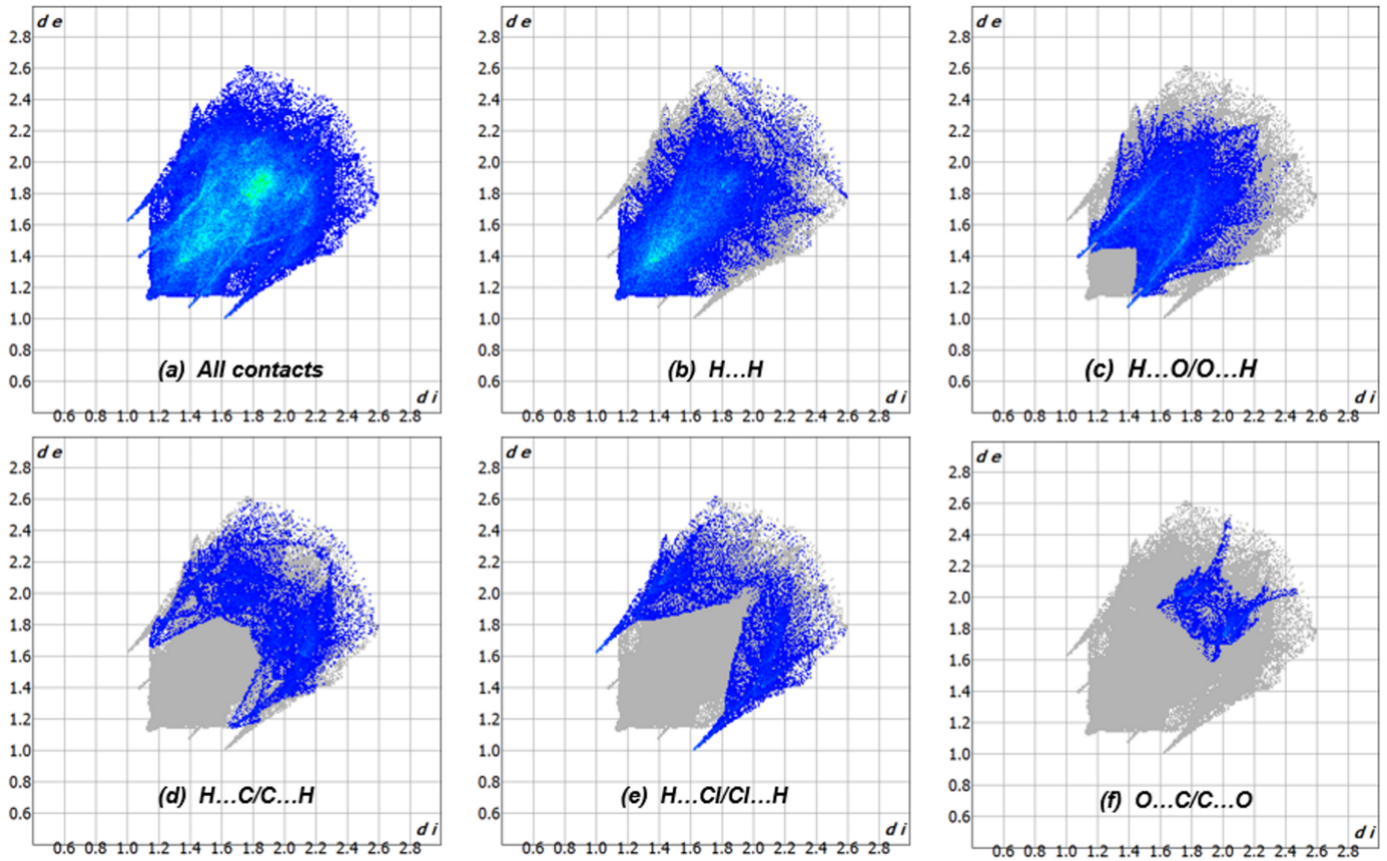
Two-dimensional fingerprint plots of the inter­mol­ecular contacts in **1**.

**Figure 5:**
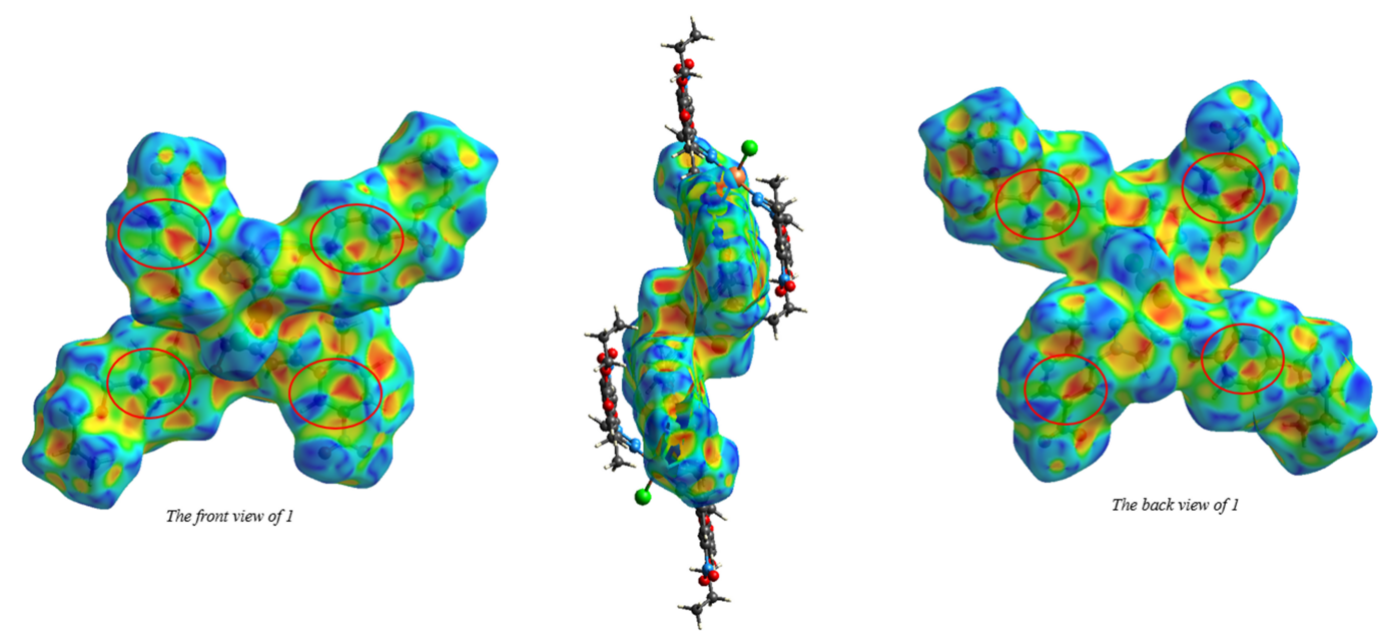
View of the Hirshfeld surface of the title compound plotted over shape-index: front and back views of middle mol­ecule, respectively.

A Hirshfeld surface analysis was performed using *CrystalExplorer21* (Spackman *et al.*, 2021 ▸). The Hirshfeld surface of mol­ecule **1** mapped over *d*_norm_ is shown in Fig. 3 ▸. Inter­mol­ecular C—H⋯O and C—H⋯Cl contacts are shown, indicating close inter­actions (hydrogen bonds) as blue and red dashed lines, respectively. The 2D fingerprint plots (McKinnon *et al.*, 2007 ▸), indicate that inter­mol­ecular H⋯H and O⋯H/H⋯O contacts make the largest contributions to the total Hirshfeld surface, 38.8% and 25.1%, respectively, with other significant contributions being H⋯C/C⋯H (10.0%), H⋯Cl/Cl⋯H (8.9%) and O⋯C/C⋯O (3.3%) (Fig. 4 ▸). The characteristic pair of spikes in the H⋯Cl/Cl⋯H and especially O⋯H/H⋯O plots shown in Fig. 4 ▸*c* and Fig. 4 ▸*e* are also indicative of hydrogen bonds. The Hirshfeld surface mapped over shape-index properties (Fig. 5 ▸) illustrates the π–π stacking interactions .

## 5. Database survey

A search of the Cambridge Structural Database (CSD, Version 5.46, November 2024; Groom *et al.*, 2016 ▸) for the generalized 4-(phen­oxy­meth­yl)-1-phenyl-1*H*-1,2,3-triazole with triazole coordination to copper returned zero relevant hits. A search instead with 4-(pyridine­sulfanylymeth­yl)-1-phenyl-1*H*-1,2,3-triazole returned one hit, CSD refcode GORBAT (Gonzalez-Silva *et al.*, 2019 ▸). Four six-coordinate examples containing two bidentate 4-(pyridine)-1-phenyl-1*H*-1,2,3-triazole moieties and two chloride ligands have been reported (CSD refcodes KINNAZ, KINNED, KINNIH, KINNON and KINNUT; Conradie *et al.*, 2018 ▸). The CSD returned less than 25 examples of four-coordinate copper(II) coordinated with exactly two chloride ligands and at least one N-coordinating triazole-derived ligand. Only eight examples have the chloride ligands in a *trans* or near-*trans* geometry, and of these, six include substituted benzotriazole ligands. The structure most similar to the title complex is bis­{4-[(benz­yloxy)meth­yl]-1-(4-chloro­benz­yl)-1*H*-1,2,3-triazole}di­chloro­copper(II) (CSD refcode QOCBAN; Mendoza-Espinosa *et al.*, 2014 ▸).

## 6. Synthesis and crystallization

The starting reagents used for the synthesis of the title coordination compound – CuCl_2_·2H_2_O (chemical grade), 2-amino­ethanol (MEA) (analytical grade) and ethyl alcohol (analytical grade) – were used as received. 40 mg (0.01 mmol) of C_20_H_20_N_4_O_5_ triazole ligand and 20 mg (0.12 mmol) of CuCl_2_·2H_2_O were added to 0.4 ml of C_2_H_7_NO and 4 ml of C_2_H_6_O solution in a glass vial. The mixture cleared and became a navy blue solution, without sediment. It was then stored in the dark at room temperature for two weeks, after which dark-pink prism-shaped crystals of the complex formed. The yield was 33 mg (55%), m.p. 491–499 K.

## 7. Refinement details

Crystal data, data collection and structure refinement details are summarized in Table 2 ▸. H atoms were positioned geometrically and refined using a riding model with distance constraints of C—H = 0.93 Å (aromatic) and 0.97 Å (methyl­ene) with *U*_iso_(H) = 1.2*U*_eq_(C); C—H = 0.96 Å (meth­yl) with *U*_iso_(H) = 1.5*U*_eq_(C).

## Supplementary Material

Crystal structure: contains datablock(s) I. DOI: 10.1107/S2056989025001732/ej2010sup1.cif

Structure factors: contains datablock(s) I. DOI: 10.1107/S2056989025001732/ej2010Isup2.hkl

CCDC reference: 2426436

Additional supporting information:  crystallographic information; 3D view; checkCIF report

## Figures and Tables

**Table 1:** Hydrogen-bond geometry (Å, °)

*D*—H⋯*A*	*D*—H	H⋯*A*	*D*⋯*A*	*D*—H⋯*A*
C7—H7⋯O9	0.93	2.39	2.706 (4)	100
C22—H22⋯Cl^i^	0.93	2.78	3.689 (3)	167
C29—H29*B*⋯O8^ii^	0.96	2.59	3.531 (6)	168

Symmetry codes: (i) 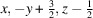; (ii) 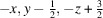.

**Table 2:** Experimental details

Crystal data	
Chemical formula	[CuCl_2_(C_20_H_20_N_4_O_5_)_2_]
*M*_r_	927.24
Crystal system, space group	Monoclinic, *P*2_1_/*c*
Temperature (K)	295
*a*, *b*, *c* (Å)	15.8765 (4), 16.2381 (3), 8.0187 (2)
β (°)	92.984 (2)
*V* (Å^3^)	2064.45 (8)
*Z*	2
Radiation type	Cu *K*α
μ (mm^−1^)	2.52
Crystal size (mm)	0.4 × 0.2 × 0.1

Data collection	
Diffractometer	XtaLAB Synergy, Single source at home/near, HyPix3000
Absorption correction	Multi-scan (*CrysAlis PRO*; Rigaku OD, 2020 ▸)
*T*_min_, *T*_max_	0.617, 1.000
No. of measured, independent and observed [*I* > 2σ(*I*)] reflections	11763, 3975, 3315
*R*_int_	0.034
(sin θ/λ)_max_ (Å^−1^)	0.615

Refinement	
*R*[*F*^2^ > 2σ(*F*^2^)], *wR*(*F*^2^), *S*	0.058, 0.171, 1.08
No. of reflections	3975
No. of parameters	279
H-atom treatment	H-atom parameters constrained
Δρ_max_, Δρ_min_ (e Å^−3^)	1.11, −0.73

Computer programs: *CrysAlis PRO* (Rigaku OD, 2020 ▸), *OLEX2.solve* (Bourhis *et al.*, 2015 ▸), *OLEX2* (Dolomanov *et al.*, 2009 ▸), *SHELXL2019/3* (Sheldrick, 2015 ▸) and *publCIF* (Westrip, 2010 ▸).

## supplementary crystallographic information

### Dichloridobis(propyl 4-{[1-(2-methyl-4-nitrophenyl)-1H-1,2,3-triazol-4-yl]methoxy}benzoate)copper(II) Crystal data

**Table:**

[CuCl_2_(C_20_H_20_N_4_O_5_)_2_]	*F*(000) = 958
*M**_r_* = 927.24	*D*_x_ = 1.492 Mg m^−^^3^
Monoclinic, *P*2_1_/*c*	Cu *K*α radiation, λ = 1.54184 Å
*a* = 15.8765 (4) Å	Cell parameters from 6038 reflections
*b* = 16.2381 (3) Å	θ = 2.8–71.2°
*c* = 8.0187 (2) Å	µ = 2.52 mm^−^^1^
β = 92.984 (2)°	*T* = 295 K
*V* = 2064.45 (8) Å^3^	Prism, metallic pinkish pink
*Z* = 2	0.4 × 0.2 × 0.1 mm

### Dichloridobis(propyl 4-{[1-(2-methyl-4-nitrophenyl)-1H-1,2,3-triazol-4-yl]methoxy}benzoate)copper(II) Data collection

**Table:**

XtaLAB Synergy, Single source at home/near, HyPix3000 diffractometer	3315 reflections with *I* > 2σ(*I*)
Detector resolution: 10.000 pixels mm^-1^	*R*_int_ = 0.034
scan	θ_max_ = 71.5°, θ_min_ = 2.8°
Absorption correction: multi-scan (CrysAlisPro; Rigaku OD, 2020)	*h* = −18→19
*T*_min_ = 0.617, *T*_max_ = 1.000	*k* = −19→16
11763 measured reflections	*l* = −9→9
3975 independent reflections	

### Dichloridobis(propyl 4-{[1-(2-methyl-4-nitrophenyl)-1H-1,2,3-triazol-4-yl]methoxy}benzoate)copper(II) Refinement

**Table:**

Refinement on *F*^2^	Primary atom site location: iterative
Least-squares matrix: full	Hydrogen site location: inferred from neighbouring sites
*R*[*F*^2^ > 2σ(*F*^2^)] = 0.058	H-atom parameters constrained
*wR*(*F*^2^) = 0.171	*w* = 1/[σ^2^(*F*_o_^2^) + (0.0806*P*)^2^ + 2.7811*P*] where *P* = (*F*_o_^2^ + 2*F*_c_^2^)/3
*S* = 1.08	(Δ/σ)_max_ < 0.001
3975 reflections	Δρ_max_ = 1.11 e Å^−^^3^
279 parameters	Δρ_min_ = −0.73 e Å^−^^3^
0 restraints	

### Dichloridobis(propyl 4-{[1-(2-methyl-4-nitrophenyl)-1H-1,2,3-triazol-4-yl]methoxy}benzoate)copper(II) Special details

**Table:**

Geometry. All esds (except the esd in the dihedral angle between two l.s. planes) are estimated using the full covariance matrix. The cell esds are taken into account individually in the estimation of esds in distances, angles and torsion angles; correlations between esds in cell parameters are only used when they are defined by crystal symmetry. An approximate (isotropic) treatment of cell esds is used for estimating esds involving l.s. planes.

### Dichloridobis(propyl 4-{[1-(2-methyl-4-nitrophenyl)-1H-1,2,3-triazol-4-yl]methoxy}benzoate)copper(II) Fractional atomic coordinates and isotropic or equivalent isotropic displacement parameters (Å^2^)

**Table:**

	*x*	*y*	*z*	*U*_iso_*/*U*_eq_	
Cu	0.500000	1.000000	0.500000	0.0332 (2)	
Cl	0.47301 (8)	0.90207 (6)	0.68768 (12)	0.0572 (3)	
N14	0.51141 (17)	0.91436 (16)	0.3218 (3)	0.0326 (6)	
N13	0.58540 (18)	0.90064 (16)	0.2623 (3)	0.0356 (6)	
N12	0.57226 (17)	0.84290 (16)	0.1438 (3)	0.0335 (6)	
C16	0.4907 (2)	0.8206 (2)	0.1295 (4)	0.0388 (7)	
H16	0.466154	0.781814	0.056789	0.047*	
C15	0.4514 (2)	0.86703 (19)	0.2442 (4)	0.0357 (7)	
C11	0.3628 (2)	0.86819 (19)	0.2929 (5)	0.0406 (8)	
H11A	0.356711	0.904300	0.387925	0.049*	
H11B	0.325842	0.887620	0.201033	0.049*	
O10	0.34212 (16)	0.78556 (14)	0.3351 (4)	0.0475 (6)	
C5	0.2652 (2)	0.7721 (2)	0.3977 (4)	0.0373 (7)	
C6	0.2435 (2)	0.6895 (2)	0.4118 (5)	0.0430 (8)	
H6	0.279257	0.648799	0.375015	0.052*	
C7	0.1692 (2)	0.6684 (2)	0.4801 (5)	0.0430 (8)	
H7	0.155728	0.613122	0.492948	0.052*	
C2	0.1138 (2)	0.7287 (2)	0.5304 (4)	0.0374 (7)	
C3	0.1353 (2)	0.8107 (2)	0.5119 (5)	0.0444 (8)	
H3	0.098309	0.851467	0.544037	0.053*	
C4	0.2108 (2)	0.8329 (2)	0.4463 (5)	0.0450 (8)	
H4	0.224769	0.888164	0.435060	0.054*	
C1	0.0341 (2)	0.7081 (2)	0.6095 (5)	0.0417 (8)	
O9	0.02717 (16)	0.62677 (16)	0.6325 (3)	0.0488 (6)	
C27	−0.0424 (2)	0.5987 (2)	0.7267 (5)	0.0469 (9)	
H27A	−0.095493	0.618928	0.677040	0.056*	
H27B	−0.036330	0.618210	0.841093	0.056*	
C28	−0.0399 (3)	0.5062 (2)	0.7216 (6)	0.0589 (11)	
H28A	−0.051299	0.488279	0.607285	0.071*	
H28B	−0.084449	0.484809	0.787746	0.071*	
C29	0.0439 (4)	0.4699 (3)	0.7865 (7)	0.0789 (16)	
H29A	0.087621	0.486837	0.715733	0.118*	
H29B	0.040059	0.410879	0.786445	0.118*	
H29C	0.056727	0.488996	0.898212	0.118*	
C17	0.6440 (2)	0.8097 (2)	0.0661 (4)	0.0342 (7)	
C22	0.6531 (2)	0.7246 (2)	0.0682 (4)	0.0358 (7)	
H22	0.613273	0.690744	0.114771	0.043*	
C21	0.7238 (2)	0.6921 (2)	−0.0018 (4)	0.0380 (7)	
C20	0.7843 (2)	0.7405 (2)	−0.0689 (5)	0.0465 (8)	
H20	0.831569	0.717168	−0.113705	0.056*	
C19	0.7731 (2)	0.8247 (2)	−0.0681 (5)	0.0467 (9)	
H19	0.813681	0.857969	−0.113702	0.056*	
C18	0.7032 (2)	0.8617 (2)	−0.0014 (4)	0.0393 (8)	
C26	0.6933 (3)	0.9537 (2)	−0.0065 (5)	0.0553 (10)	
H26A	0.636487	0.967348	−0.043287	0.083*	
H26B	0.731712	0.976626	−0.082638	0.083*	
H26C	0.705328	0.975968	0.103051	0.083*	
N23	0.7348 (2)	0.60191 (19)	0.0011 (4)	0.0469 (8)	
O25	0.6897 (2)	0.56138 (16)	0.0887 (4)	0.0610 (8)	
O24	0.7876 (2)	0.5722 (2)	−0.0855 (5)	0.0753 (10)	
O8	−0.01809 (19)	0.75633 (18)	0.6508 (5)	0.0674 (9)	

### Dichloridobis(propyl 4-{[1-(2-methyl-4-nitrophenyl)-1H-1,2,3-triazol-4-yl]methoxy}benzoate)copper(II) Atomic displacement parameters (Å^2^)

**Table:**

	*U*^11^	*U*^22^	*U*^33^	*U*^12^	*U*^13^	*U*^23^
Cu	0.0392 (4)	0.0267 (3)	0.0345 (4)	−0.0014 (3)	0.0092 (3)	−0.0058 (3)
Cl	0.0881 (8)	0.0353 (5)	0.0505 (5)	0.0033 (4)	0.0254 (5)	0.0041 (4)
N14	0.0386 (14)	0.0267 (13)	0.0330 (13)	−0.0035 (11)	0.0078 (11)	−0.0039 (10)
N13	0.0396 (15)	0.0324 (14)	0.0351 (14)	−0.0030 (12)	0.0053 (11)	−0.0094 (11)
N12	0.0409 (15)	0.0262 (12)	0.0339 (14)	−0.0016 (11)	0.0074 (11)	−0.0061 (10)
C16	0.0420 (18)	0.0297 (16)	0.0449 (18)	−0.0054 (14)	0.0030 (14)	−0.0080 (14)
C15	0.0413 (18)	0.0257 (15)	0.0403 (17)	−0.0024 (13)	0.0038 (14)	−0.0013 (13)
C11	0.0394 (18)	0.0260 (16)	0.057 (2)	−0.0019 (13)	0.0066 (15)	−0.0025 (15)
O10	0.0418 (13)	0.0270 (11)	0.0755 (18)	−0.0004 (10)	0.0185 (12)	0.0026 (11)
C5	0.0350 (17)	0.0317 (16)	0.0452 (18)	−0.0029 (13)	0.0020 (14)	0.0017 (14)
C6	0.0430 (19)	0.0295 (16)	0.057 (2)	0.0009 (14)	0.0102 (16)	−0.0007 (15)
C7	0.0456 (19)	0.0302 (17)	0.054 (2)	−0.0036 (15)	0.0050 (16)	0.0018 (15)
C2	0.0355 (17)	0.0356 (17)	0.0410 (18)	−0.0031 (14)	0.0002 (13)	0.0017 (14)
C3	0.0407 (19)	0.0318 (17)	0.061 (2)	0.0032 (15)	0.0078 (16)	−0.0012 (16)
C4	0.044 (2)	0.0292 (16)	0.063 (2)	−0.0011 (15)	0.0116 (17)	0.0023 (16)
C1	0.0415 (19)	0.0403 (18)	0.0433 (18)	−0.0015 (15)	0.0022 (15)	0.0019 (15)
O9	0.0464 (14)	0.0397 (13)	0.0622 (16)	−0.0055 (11)	0.0210 (12)	0.0031 (12)
C27	0.0420 (19)	0.052 (2)	0.048 (2)	−0.0043 (17)	0.0133 (16)	0.0029 (17)
C28	0.070 (3)	0.049 (2)	0.060 (3)	−0.022 (2)	0.027 (2)	−0.0047 (19)
C29	0.093 (4)	0.051 (3)	0.096 (4)	0.016 (3)	0.038 (3)	0.017 (3)
C17	0.0398 (17)	0.0303 (16)	0.0330 (16)	0.0001 (13)	0.0057 (13)	−0.0040 (13)
C22	0.0433 (18)	0.0307 (16)	0.0336 (16)	−0.0003 (14)	0.0043 (13)	−0.0019 (13)
C21	0.0429 (18)	0.0351 (17)	0.0357 (17)	0.0060 (14)	−0.0005 (14)	−0.0031 (14)
C20	0.0413 (19)	0.051 (2)	0.048 (2)	0.0038 (17)	0.0066 (15)	−0.0079 (17)
C19	0.046 (2)	0.051 (2)	0.044 (2)	−0.0079 (17)	0.0121 (16)	−0.0016 (16)
C18	0.051 (2)	0.0346 (17)	0.0329 (16)	−0.0049 (15)	0.0057 (14)	−0.0013 (13)
C26	0.078 (3)	0.0349 (19)	0.055 (2)	−0.0079 (19)	0.020 (2)	0.0034 (17)
N23	0.0530 (19)	0.0382 (16)	0.0492 (18)	0.0113 (14)	−0.0018 (15)	−0.0043 (14)
O25	0.093 (2)	0.0366 (14)	0.0546 (17)	0.0082 (14)	0.0110 (15)	0.0061 (12)
O24	0.064 (2)	0.0563 (18)	0.107 (3)	0.0209 (16)	0.0236 (18)	−0.0154 (18)
O8	0.0484 (17)	0.0448 (16)	0.111 (3)	0.0069 (13)	0.0250 (16)	0.0018 (16)

### Dichloridobis(propyl 4-{[1-(2-methyl-4-nitrophenyl)-1H-1,2,3-triazol-4-yl]methoxy}benzoate)copper(II) Geometric parameters (Å, º)

**Table:**

Cu—Cl	2.2460 (9)	C1—O8	1.199 (5)
Cu—Cl^i^	2.2460 (9)	O9—C27	1.444 (4)
Cu—N14^i^	2.009 (2)	C27—H27A	0.9700
Cu—N14	2.009 (2)	C27—H27B	0.9700
N14—N13	1.310 (4)	C27—C28	1.503 (5)
N14—C15	1.350 (4)	C28—H28A	0.9700
N13—N12	1.344 (4)	C28—H28B	0.9700
N12—C16	1.344 (4)	C28—C29	1.522 (7)
N12—C17	1.432 (4)	C29—H29A	0.9600
C16—H16	0.9300	C29—H29B	0.9600
C16—C15	1.365 (5)	C29—H29C	0.9600
C15—C11	1.481 (5)	C17—C22	1.390 (4)
C11—H11A	0.9700	C17—C18	1.394 (5)
C11—H11B	0.9700	C22—H22	0.9300
C11—O10	1.426 (4)	C22—C21	1.385 (5)
O10—C5	1.361 (4)	C21—C20	1.373 (5)
C5—C6	1.390 (5)	C21—N23	1.475 (4)
C5—C4	1.383 (5)	C20—H20	0.9300
C6—H6	0.9300	C20—C19	1.378 (5)
C6—C7	1.369 (5)	C19—H19	0.9300
C7—H7	0.9300	C19—C18	1.393 (5)
C7—C2	1.391 (5)	C18—C26	1.502 (5)
C2—C3	1.384 (5)	C26—H26A	0.9600
C2—C1	1.483 (5)	C26—H26B	0.9600
C3—H3	0.9300	C26—H26C	0.9600
C3—C4	1.381 (5)	N23—O25	1.221 (4)
C4—H4	0.9300	N23—O24	1.216 (4)
C1—O9	1.338 (4)		
			
Cl^i^—Cu—Cl	180.0	C1—O9—C27	117.0 (3)
N14^i^—Cu—Cl^i^	90.80 (8)	O9—C27—H27A	110.5
N14—Cu—Cl^i^	89.20 (8)	O9—C27—H27B	110.5
N14^i^—Cu—Cl	89.20 (8)	O9—C27—C28	106.2 (3)
N14—Cu—Cl	90.80 (8)	H27A—C27—H27B	108.7
N14^i^—Cu—N14	180.0	C28—C27—H27A	110.5
N13—N14—Cu	119.5 (2)	C28—C27—H27B	110.5
N13—N14—C15	111.1 (3)	C27—C28—H28A	108.8
C15—N14—Cu	129.4 (2)	C27—C28—H28B	108.8
N14—N13—N12	105.5 (2)	C27—C28—C29	113.7 (4)
N13—N12—C16	111.2 (3)	H28A—C28—H28B	107.7
N13—N12—C17	118.2 (3)	C29—C28—H28A	108.8
C16—N12—C17	130.3 (3)	C29—C28—H28B	108.8
N12—C16—H16	127.3	C28—C29—H29A	109.5
N12—C16—C15	105.4 (3)	C28—C29—H29B	109.5
C15—C16—H16	127.3	C28—C29—H29C	109.5
N14—C15—C16	106.8 (3)	H29A—C29—H29B	109.5
N14—C15—C11	121.9 (3)	H29A—C29—H29C	109.5
C16—C15—C11	131.2 (3)	H29B—C29—H29C	109.5
C15—C11—H11A	110.4	C22—C17—N12	116.9 (3)
C15—C11—H11B	110.4	C22—C17—C18	122.4 (3)
H11A—C11—H11B	108.6	C18—C17—N12	120.6 (3)
O10—C11—C15	106.5 (3)	C17—C22—H22	121.3
O10—C11—H11A	110.4	C21—C22—C17	117.3 (3)
O10—C11—H11B	110.4	C21—C22—H22	121.3
C5—O10—C11	117.4 (3)	C22—C21—N23	118.0 (3)
O10—C5—C6	114.6 (3)	C20—C21—C22	122.6 (3)
O10—C5—C4	125.1 (3)	C20—C21—N23	119.3 (3)
C4—C5—C6	120.3 (3)	C21—C20—H20	120.9
C5—C6—H6	120.1	C21—C20—C19	118.2 (3)
C7—C6—C5	119.8 (3)	C19—C20—H20	120.9
C7—C6—H6	120.1	C20—C19—H19	118.8
C6—C7—H7	119.6	C20—C19—C18	122.4 (3)
C6—C7—C2	120.7 (3)	C18—C19—H19	118.8
C2—C7—H7	119.6	C17—C18—C26	122.8 (3)
C7—C2—C1	122.1 (3)	C19—C18—C17	117.0 (3)
C3—C2—C7	118.8 (3)	C19—C18—C26	120.2 (3)
C3—C2—C1	119.0 (3)	C18—C26—H26A	109.5
C2—C3—H3	119.5	C18—C26—H26B	109.5
C4—C3—C2	121.1 (3)	C18—C26—H26C	109.5
C4—C3—H3	119.5	H26A—C26—H26B	109.5
C5—C4—H4	120.4	H26A—C26—H26C	109.5
C3—C4—C5	119.3 (3)	H26B—C26—H26C	109.5
C3—C4—H4	120.4	O25—N23—C21	118.1 (3)
O9—C1—C2	111.1 (3)	O24—N23—C21	118.0 (3)
O8—C1—C2	126.0 (3)	O24—N23—O25	123.9 (3)
O8—C1—O9	122.9 (3)		
			
Cu—N14—N13—N12	−178.21 (19)	C6—C7—C2—C1	−178.1 (3)
Cu—N14—C15—C16	178.1 (2)	C7—C2—C3—C4	−0.7 (6)
Cu—N14—C15—C11	−4.7 (5)	C7—C2—C1—O9	3.6 (5)
N14—N13—N12—C16	−0.1 (3)	C7—C2—C1—O8	−177.3 (4)
N14—N13—N12—C17	−174.4 (3)	C2—C3—C4—C5	0.4 (6)
N14—C15—C11—O10	−122.9 (3)	C2—C1—O9—C27	172.7 (3)
N13—N14—C15—C16	0.2 (4)	C3—C2—C1—O9	−173.9 (3)
N13—N14—C15—C11	177.5 (3)	C3—C2—C1—O8	5.2 (6)
N13—N12—C16—C15	0.2 (4)	C4—C5—C6—C7	−2.5 (6)
N13—N12—C17—C22	125.8 (3)	C1—C2—C3—C4	176.9 (3)
N13—N12—C17—C18	−51.5 (4)	C1—O9—C27—C28	175.3 (3)
N12—C16—C15—N14	−0.3 (4)	O9—C27—C28—C29	56.2 (5)
N12—C16—C15—C11	−177.1 (3)	C17—N12—C16—C15	173.7 (3)
N12—C17—C22—C21	−177.9 (3)	C17—C22—C21—C20	1.1 (5)
N12—C17—C18—C19	177.2 (3)	C17—C22—C21—N23	179.3 (3)
N12—C17—C18—C26	−3.8 (5)	C22—C17—C18—C19	0.1 (5)
C16—N12—C17—C22	−47.3 (5)	C22—C17—C18—C26	179.1 (3)
C16—N12—C17—C18	135.4 (4)	C22—C21—C20—C19	−0.9 (5)
C16—C15—C11—O10	53.6 (5)	C22—C21—N23—O25	−12.1 (5)
C15—N14—N13—N12	−0.1 (3)	C22—C21—N23—O24	167.0 (3)
C15—C11—O10—C5	174.7 (3)	C21—C20—C19—C18	0.3 (6)
C11—O10—C5—C6	170.4 (3)	C20—C21—N23—O25	166.1 (3)
C11—O10—C5—C4	−10.0 (5)	C20—C21—N23—O24	−14.8 (5)
O10—C5—C6—C7	177.1 (3)	C20—C19—C18—C17	0.1 (5)
O10—C5—C4—C3	−178.4 (4)	C20—C19—C18—C26	−178.9 (4)
C5—C6—C7—C2	2.2 (6)	C18—C17—C22—C21	−0.7 (5)
C6—C5—C4—C3	1.2 (6)	N23—C21—C20—C19	−179.1 (3)
C6—C7—C2—C3	−0.6 (6)	O8—C1—O9—C27	−6.4 (6)

Symmetry code: (i) −*x*+1, −*y*+2, −*z*+1.

### Dichloridobis(propyl 4-{[1-(2-methyl-4-nitrophenyl)-1H-1,2,3-triazol-4-yl]methoxy}benzoate)copper(II) Hydrogen-bond geometry (Å, º)

**Table:**

*D*—H···*A*	*D*—H	H···*A*	*D*···*A*	*D*—H···*A*
C7—H7···O9	0.93	2.39	2.706 (4)	100
C22—H22···Cl^ii^	0.93	2.78	3.689 (3)	167
C29—H29*B*···O8^iii^	0.96	2.59	3.531 (6)	168

Symmetry codes: (ii) *x*, −*y*+3/2, *z*−1/2; (iii) −*x*, *y*−1/2, −*z*+3/2.
